# The effect of care provided by paediatric critical care transport teams on mortality of children transported to paediatric intensive care units in England and Wales: a retrospective cohort study

**DOI:** 10.1186/s12887-021-02689-x

**Published:** 2021-05-03

**Authors:** Sarah E. Seaton, Elizabeth S. Draper, Christina Pagel, Fatemah Rajah, Jo Wray, Padmanabhan Ramnarayan, on behalf of the DEPICT Study Team, Victoria Barber, Robert Darnell, Patrick Davies, Laura Drikite, Matthew Entwistle, Ruth Evans, Emma Hudson, Enoch Kung, Will Marriage, Stephen Morris, Paul Mouncey, Anna Pearce, Eithne Polke, Elizabeth S. Draper, Christina Pagel, Fatemah Rajah, Padmanabhan Ramnarayan, Sarah E. Seaton, Jo Wray

**Affiliations:** 1 Department of Health Sciences, University of Leicester, Leicester, LE1 7RH UK; 2 Clinical Operational Research Unit, University College London, London, UK; 3 Yorkshire and Humber Infant and Children’s Transport Service (Embrace), Barnsley, UK; 4 Heart and Lung Directorate, Great Ormond Street Hospital for Children NHS Foundation Trust, London, UK; 5 Children’s Acute Transport Service (CATS), Great Ormond Street Hospital NHS Foundation Trust, London, UK; 6 Respiratory, Critical Care and Anaesthesia Section, Infection, Immunity and Inflammation Research & Teaching Department, UCL GOS Institute of Child Health, London, UK

**Keywords:** Paediatric intensive care, Paediatric transport, Critical care transport

## Abstract

**Background:**

Centralisation of paediatric intensive care units (PICUs) has the increased the need for specialist paediatric critical care transport teams (PCCT) to transport critically ill children to PICU. We investigated the impact of care provided by PCCTs for children on mortality and other clinically important outcomes.

**Methods:**

We analysed linked national data from the Paediatric Intensive Care Audit Network (PICANet) from children admitted to PICUs in England and Wales (2014–2016) to assess the impact of who led the child’s transport, whether prolonged stabilisation by the PCCT was detrimental and the impact of critical incidents during transport on patient outcome. We used logistic regression models to estimate the adjusted odds and probability of mortality within 30 days of admission to PICU (primary outcome) and negative binomial models to investigate length of stay (LOS) and length of invasive ventilation (LOV).

**Results:**

The study included 9112 children transported to PICU. The most common diagnosis was respiratory problems; junior doctors led the PCCT in just over half of all transports; and the 30-day mortality was 7.1%. Transports led by Advanced Nurse Practitioners and Junior Doctors had similar outcomes (adjusted mortality ANP: 0.035 versus Junior Doctor: 0.038). Prolonged stabilisation by the PCCT was possibly associated with increased mortality (0.059, 95% CI: 0.040 to 0.079 versus short stabilisation 0.044, 95% CI: 0.039 to 0.048). Critical incidents involving the child increased the adjusted odds of mortality within 30 days (odds ratio: 3.07).

**Conclusions:**

Variations in team composition between PCCTs appear to have little effect on patient outcomes. We believe differences in stabilisation approaches are due to residual confounding. Our finding that critical incidents were associated with worse outcomes indicates that safety during critical care transport is an important area for future quality improvement work.

**Supplementary Information:**

The online version contains supplementary material available at 10.1186/s12887-021-02689-x.

## Introduction

Paediatric Critical Care Transport (PCCT) teams were developed following the centralisation of Paediatric Intensive Care Units (PICU) in the United Kingdom and in other parts of the world [1–4]. PCCTs provide ‘intensive care on the move’ for critically ill children who require transport from general hospitals to PICUs – thus, the arrival of the PCCT at the bedside of the child represents the first contact with an intensive care team. Currently, approximately one third to half of admissions to the 24 National Health Service (NHS) PICUs in England and Wales are for children transported by PCCTs [5].

The nine PCCTs within England and Wales each serve different populations and different geographies, and have evolved over the years in terms of their service models [6]. The Paediatric Intensive Care Audit Network (PICANet), the national clinical audit of paediatric intensive care activity, reports considerable variation between PCCTs in terms of the time taken to reach a child’s bedside after agreeing the child requires intensive care, their team composition, the number and nature of interventions performed and the rate of critical incidents during transport [7]. National quality standards exist for timeliness [8], stating PCCTs should be at the child’s bedside within three hours of agreeing the child requires paediatric intensive care, but not for other aspects of care provided by the PCCTs.

The DEPICT Study (Differences in access to Emergency Paediatric Intensive Care and care during Transport) is a national mixed-methods study investigating the impact of transport to PICU on outcomes and experiences of critically ill children and their families [9]. As part of DEPICT, we previously investigated the impact of the time taken by PCCTs to reach the bedside of critically ill children and concluded that the time does not appear to be associated with mortality [10]. In this paper, we investigate the impact of care provided by the transport team for critically ill children on their 30-day mortality (primary outcome) and other clinically important outcomes.

## Methods

### Study population

The DEPICT Study includes critically ill children (aged < 16 years) transported as an emergency (non-elective) to a National Health Service (NHS) PICU in England and Wales from 1st January 2014 to 31 December 2016.

### Outcomes

The primary outcome of interest was mortality within 30 days of admission to PICU. Secondary mortality endpoints were mortality whilst in the PICU and within 90 days of admission. Secondary outcomes related to healthcare utilisation were length of PICU stay (LOS) and length of invasive ventilation in PICU (LOV).

### Data sources

Information about children transported by a PCCT to PICU were extracted from the Paediatric Intensive Care Audit Network (PICANet, https://www.picanet.org.uk/) which collects data related to the referral, transport and admission of every child requiring admission to a PICU. Data entry into PICANet within three months of discharge is recommended by standards set by the Paediatric Intensive Care Society (PICS) [8], and data completeness, including NHS number which was used for the data linkage, is around 99% [7]. PICANet uses a bespoke web-based data entry system supplemented by regular feedback to units and validation visits to ensure data are accurately entered from medical notes.

Information about admissions to a general (adult) intensive care unit (GICU) prior to transport to a PICU was provided by the Intensive Care National Audit and Research Centre (ICNARC) and linked to PICANet data using personally identifiable data by NHS Digital (https://digital.nhs.uk/). Mortality outcomes were provided from the Office for National Statistics. Further details about the data flow and linkage can be found in the DEPICT Study protocol [9].

### Inclusion and exclusion criteria

Children were included if their PICANet transport record linked to a corresponding PICU admission. Children with missing referral data were excluded. If a child was transported multiple times during DEPICT we only included their final transport. Children were also excluded if there was missing information about ventilation status at referral, or if they had missing or implausible time data (defined as > 24 h) for the time-to-bedside; time spent at the bedside or the total time taken to reach the PICU. In the analysis of team composition children were excluded if they had missing data about the team leader of the transport. In the analysis of secondary healthcare outcomes, children were excluded if they had missing data about LOS or LOV.

### Statistical analysis

Summary statistics were reported as counts/percentage for categorical variables and median/range for continuous variables. Adjustments throughout our work were selected a priori, before any analysis, by clinical members of the Study Management Group: time to reach the bedside; age of the child; severity of illness measured by the Paediatric Index of Mortality 2 (PIM2) score [11]; clinical diagnosis; ventilation status at the time of referral and whether the child was receiving critical care around the time of the transport request. All variables were included regardless of their statistical significance or their impact on the outcome. Statistical significance is not reported in line with the DEPICT Study protocol and emphasis is on trends and clinical relevance of findings.

### Team leader

Transport team leaders were from one of three grades of staff: Junior Doctor (doctor in training – fellow); Advanced Nurse Practitioner (ANP) or a Consultant (attending physician). We undertook three comparisons regarding the team leader using logistic regression models with mortality as the outcome: (1) Consultant versus not a Consultant; (2) Junior Doctor versus ANP and (3) all three options. Information is not collected in the data about the rationale for the selection of the team leader, although Consultants are often used for the most critical cases. Not all regions of England and Wales have access to ANPs.

### Prolonged stabilisation by the PCCT versus short stabilisation

We focused on key clinical interventions provided to the child, including: intubation and re-intubation (airway procedures), central venous access, arterial access and intraosseous access (vascular access procedures) and initiation of vasoactive infusions. Clinical interventions were provided by the referring hospital prior to the PCCT arrival, or provided when the PCCT was in attendance. Firstly we fitted logistic regression models to compare the scenario where the PCCT spent substantial time preparing the child for transport (prolonged stabilisation: two or more interventions provided whilst the PCCT were in attendance) versus short stabilisation (< 2 interventions provided by the PCCT). For outcomes related to mortality we used logistic regression models and for LOS and LOV we used negative binomial models. We adjusted for: age; PIM2 score; diagnosis of the child; whether the child was ventilated at the time of referral; whether the child was receiving critical care and the time taken to reach the bedside.

### Number and types of interventions

We investigated the impact of the total number of interventions received by the child and the percentage of interventions which were provided by the PCCT using logistic regression models. We also considered the impact of interventions on our secondary outcomes of LOS and LOV via use of negative binomial models. We adjusted for: age; PIM2 score; diagnosis of the child; whether the child was ventilated at the time of referral; whether the child was receiving critical care and the time taken to reach the bedside. To investigate the impact that specific interventions had on stabilisation time we fitted a linear regression model with time spent stabilising the child (in minutes) as the outcome.

We investigated whether there was an impact on mortality related to who (referring hospital or PCCT) initiated the provision of certain interventions (grouped as provision of: airway procedures; vascular access procedures and vasoactive infusions).

### Critical incidents

Finally, we investigated instances of critical incidents that occurred during the transport involving either the child, vehicle or an equipment failure impacting on the child’s care and whether these impacted on the adjusted odds of mortality. Critical incidents involving the child were: accidental extubation; required intubation in transit; complete ventilator failure; loss of medical gas supply; loss of all IV access; cardiac arrest and medication administration error. Vehicle incidents included: accidents and breakdown. We adjusted for: age; PIM2 score; diagnosis of the child; whether the child was ventilated at the time of referral; whether the child was receiving critical care and the time taken to reach the bedside.

### Ethical approval

DEPICT has ethical approval from the National Research Ethics Service (London Riverside, reference: 17/LO/1267) and agreement from the Confidentiality Advisory Group (reference: CAG0129) to use data collected without patient consent. The study followed all relevant guidelines and regulations (Declaration of Helsinki).

### Role of the funding source

The DEPICT Study is funded by the National Institute for Health Research (NIHR) Health Services and Delivery Research (reference: 15/136/45). The views expressed in this work are those of the author(s) and not necessarily those of the NIHR or the Department of Health and Social Care.

## Results

### Study population

There were 10,987 emergency transports by a PCCT of children aged under 16 years with a linked admission record to a PICU during the study (Additional file 1: Figure 1). Linkage between PICANet transport and admission records was very high (~ 97%). Transports not linked with a corresponding referral event were excluded (*n* = 471, 4.3%) leaving 10,516 transports. For children with multiple transports we used the latest transport, providing 9438 transported children. Children whose ventilation status at the time of referral was missing (*n* = 272) and those with missing or implausible data (defined as > 24 h) for the time-to-bedside (*n* = 50) or time-to-PICU (n = 2) or a missing stabilisation time (n = 2) were excluded. A total of 9112 children were included in the analysis. Children were excluded from subsequent analyses if they had missing data about LOS (*n* = 0); LOV (*n* = 1) or the grade of the team leader undertaking the transport (*n* = 16).

Summary statistics concerning the children included in this work are provided in Table 1. Over half of the children were aged under one year at the time of transport and the most common clinical diagnoses related to respiratory problems. There was a positive relationship between the number of interventions delivered by the PCCT and the time spent stabilising the child before transport. Over half of children were transported with a Junior Doctor in charge of the transport and 5.4% reported a critical incident involving the child, vehicle or equipment.

**Table 1:** Summary statistics for the children included in the analysis

Characteristics	Total (***n*** = 9112)
**Age of the child, n (%)**	
0 to < 1 year	4668 (51.2)
1 to < 5 years	2436 (26.7)
5 to < 11 years	1174 (12.9)
11 to < 16 years	834 (9.2)
**Sex of child, n (%)**	
Male	5181 (56.7)
Female	3930 (43.1)
Unknown	1 (< 0.1)
**PIM2 group, n (%)**	
< 1%	1039 (11.4)
1 to < 5%	4087 (44.9)
5 to < 15%	2983 (32.7)
15 to < 30%	579 (6.4)
30 + %	424 (4.7)
**Diagnosis of the child, n (%)**	
Cardiovascular	1309 (14.4)
Endocrine	219 (2.4)
Haem/oncology	153 (1.7)
Infection	820 (9.0)
Neurological	1504 (16.5)
Trauma and accidents	338 (3.7)
Respiratory	4353 (47.8)
Other	416 (4.6)
**Time to bedside (minutes), median (10th, 90th centile)**	83 (42 to 208)
**Time at bedside (minutes), median (10th, 90th centile)**	105 (56 to 191)
**Journey time to PICU (minutes), median (10th, 90th centile)**	50 (25 to 100)
**Stabilisation time by number of interventions delivered by PCCT, median (10th, 90th centile)**	
0	90 (50 to 150)
1	125 (80 to 200)
2	157 (100 to 241)
3	180 (110 to 279)
4+	207 (135 to 315)
**Grade of team leader, n (%)**	
Consultant	3028 (33.2)
Junior Doctor	4726 (51.9)
Advanced Nurse Practitioner	1342 (14.7)
Unknown	16 (0.2)
**Stabilisation approach**	
Short (< 2 interventions by PCCT)	7830 (85.9)
Prolonged (≥2 interventions by PCCT)	1282 (14.1)
**Critical incidents, n (%)**	
Child incident	121 (1.3)
Vehicle incident	55 (0.6)
Equipment failure	333 (3.7)
Any incident	496 (5.4)
**Died in two days of admission to PICU, n (%)**	278 (3.1)
**Died in PICU, n (%)**	571 (6.3)
**Died in thirty days of admission to PICU, n (%)**	645 (7.1)
**Died in one year of admission to PICU, n (%)**	949 (10.4)

### Team leader

**Fig. 1:**
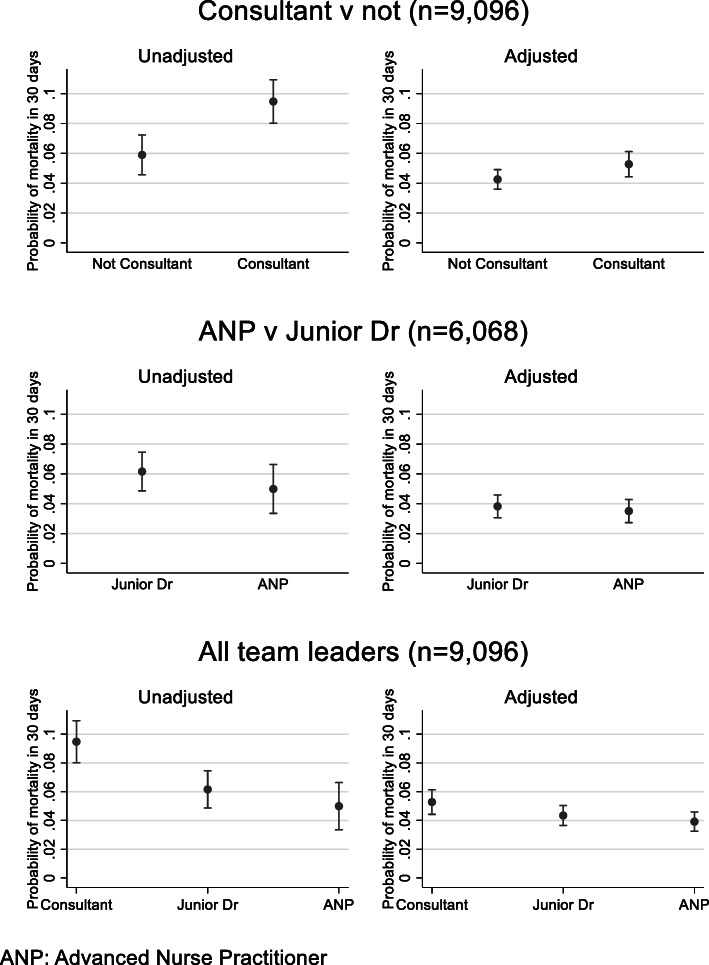
Grade of the team leader of the transport and adjusted mortality within 30 days of admission to PICU. Adjusted probabilities are estimated whilst holding other covariates at their average value. Adjustments were made for: time taken to reach the bedside of the child; age of child; PIM 2 score; diagnosis of the child; whether they were ventilated at the time of referral and whether they were receiving critical care

All regions of the country have access to a Consultant or Junior Doctor, but not every region has access to an ANP. Before adjustment, Consultant-led transports had the highest probability of mortality and after adjustment, they still had the highest mortality although the difference was substantially diminished. There were no differences in the adjusted mortality between transports led by ANPs and Junior Doctors (ANP: 0.035 versus Junior Doctor: 0.038, Fig. 1).

### Stabilisation models

For the primary outcome, there was a marked difference in the unadjusted mortality between children who had received prolonged stabilisation and those who had not (0.137 versus 0.060, Table 2). After adjustment, the difference was reduced substantially (0.059 versus 0.044, Table 2), indicating that PCCTs were potentially providing more interventions to sicker children and our case mix adjustment accounted for the majority of the variation, although a difference did remain. Differences were seen in mortality at other time points between the children who had received prolonged stabilisation and those who had not, but again differences were reduced markedly in our adjustment (Table 2). However, differences were more apparent in LOS and LOV, where even after adjustment differences of more than one day were noted between the two groups of children.

**Table 2:** Unadjusted and adjusted mortality comparing children transported following prolonged stabilisation from the PCCT (≥2 interventions conducted by the PCCT) versus short stabilisation (< 2 interventions performed by PCCT). Adjusted probabilities are estimated whilst holding other covariates at their average value

	**Unadjusted probability**	**95% confidence interval**	**Adjusted**^a^ **probability**	**95% confidence interval**
**Mortality in 30 days**				
Short stabilisation	0.060	0.051 to 0.069	0.044	0.039 to 0.048
Prolonged stabilisation	0.137	0.122 to 0.151	0.059	0.040 to 0.079
**Mortality in PICU**				
Short stabilisation	0.051	0.041 to 0.061	0.035	0.030 to 0.039
Prolonged stabilisation	0.135	0.123 to 0.147	0.056	0.036 to 0.076
**Mortality in 90 days**				
Short stabilisation	0.075	0.063 to 0.087	0.059	0.052 to 0.066
Prolonged stabilisation	0.158	0.143 to 0.174	0.079	0.055 to 0.103
	**Unadjusted expected number of days**	**95% confidence interval**	**Adjusted**^**a**^ **expected number of days**	**95% confidence interval**
**Length of stay**				
Short stabilisation	7.28	6.63 to 7.93	7.04	6.65 to 7.42
Prolonged stabilisation	9.15	8.12 to 10.19	8.47	7.56 to 9.39
**Length of ventilation**				
Short stabilisation	5.09	4.68 to 5.50	4.84	4.53 to 5.15
Prolonged stabilisation	6.74	5.88 to 7.59	6.18	5.33 to 7.02

^a^Adjustments made for: age; PIM2 score; diagnosis of the child; whether the child was ventilated at the time of referral; whether the child was receiving critical care and the time taken to reach the bedside. Cluster term included for the PCCT

As the number of interventions provided by the PCCT increased so did the median stabilisation time (Table 1). To explore the impact of prolonged stabilisation time further, the time spent stabilising the child according to the provision of different clinical interventions by the PCCT, after adjusting for child level characteristics, can be found in Additional file 1: Table 1. For example, stabilisation time was 36 min longer for children who were intubated whilst the PCCT were in attendance compared to those who were not.

Irrespective of whether children received prolonged stabilisation by the PCCT or not, the number of interventions provided by the referring hospital was similar in both groups of children (median: 1 vs 1 in prolonged versus short stabilisation, mean: 1.5 versus 1.3 in prolonged versus short stabilisation). As the time taken to reach the bedside of the child increased, the median number of interventions delivered by the referring hospital remained similar.

### Number and types of clinical interventions

**Fig. 2:**
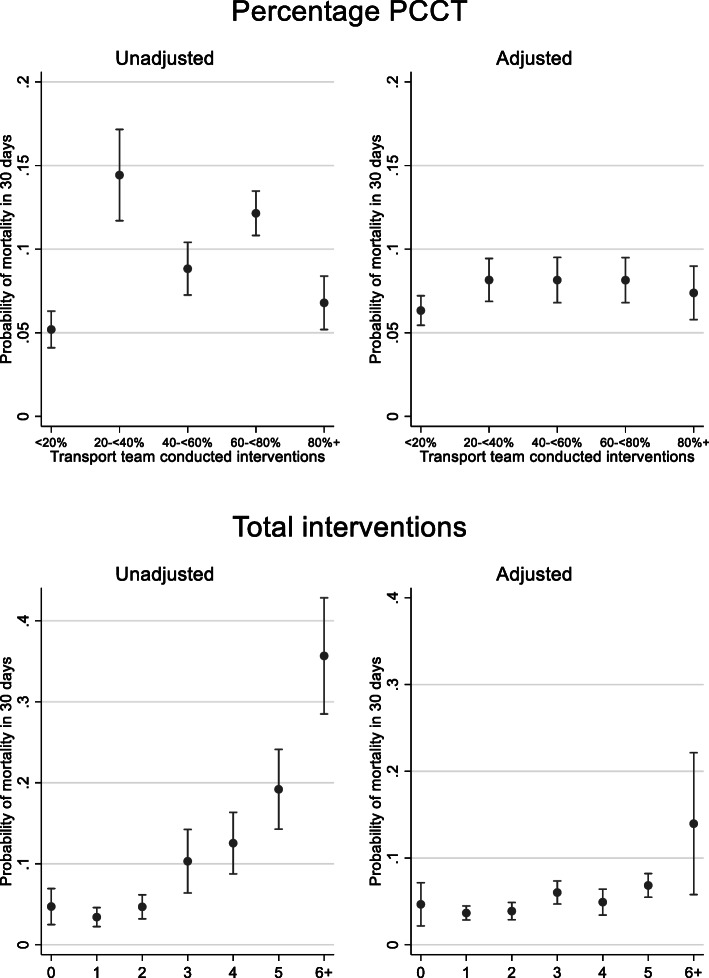
Unadjusted and adjusted mortality by the percentage of interventions delivered whilst the PCCT were present and in total. Adjusted probabilities are estimated whilst holding other covariates at their average value. Adjustments were made for: time taken to reach the bedside of the child; age of child; PIM 2 score; diagnosis of the child; whether they were ventilated at the time of referral and whether they were receiving critical care

We considered the percentage of interventions conducted by the PCCT and the total number of interventions received by the child (Fig. 2). There appeared to be no relationship in the unadjusted or adjusted analysis between the percentage of interventions that were delivered by the PCCT and mortality. When considering the total number of interventions provided to the child both by the referring hospital and the PCCT, the unadjusted probability of mortality at 30 days increased markedly as the number of interventions increased (Fig. 2). After adjustment, the trend was markedly diminished although there was still an increase in mortality in the children receiving the most interventions, again potentially indicating that the number of interventions was another proxy of the sickness of the child not captured by the other variables. Similar relationships were seen for other mortality endpoints (not shown). For our secondary outcomes, both LOS and LOV demonstrated a weak increasing relationship as the percentage of interventions delivered by the PCCT increased. There was an increase in LOS and LOV as the total number of interventions increased (Additional file 1: Figure 2a and 2b).

We considered whether there was a difference if specific interventions were initiated by the referring hospital or the PCCT (Additional file 1: Table 2). After adjustment, the highest odds of mortality was for children who have vasoactive infusions initiated by the local team compared to children who did not require vasoactive infusions (odds ratio: 1.67, 95% CI: 1.37 to 2.05). Children also had elevated odds of mortality if they had vascular access procedures provided by the referring hospital or the PCCT compared to those children who did not have vascular access (ORs: 1.38 and 1.20, Additional file 1: Table 2). However, confidence intervals for the PCCT and local team both spanned one (the point of no difference) and the odds ratios were elevated in both groups similarly.

### Critical incidents

Finally, we considered the impact of critical incidents on the child’s outcome (Table 3). The adjusted odds of 30 day mortality for all incidents was 1.60 (95% CI: 1.05 to 2.45) compared to children who did not experience an incident. Incidents involving the child had the highest adjusted odds of mortality (odds ratio: 3.07, 95% CI: 1.48 to 6.35), although elevated odds were also noted for equipment failure (odds ratio: 1.15, 95% CI: 0.75 to 1.74). Incidents involving the vehicle resulted in reduced adjusted odds of mortality (odds ratio: 0.47, 95% CI: 0.23 to 0.96), but these incidents were uncommon (*n* = 55, Table 1).

**Table 3:** Unadjusted and adjusted odds ratios of mortality in 30 days of admission to PICU by category of critical incidents

	Unadjusted odds ratio	95% confidence interval	Adjusted^a^ odds ratio	95% confidence intervals
**Any critical incident (baseline: no incident)**	1.96	1.46 to 2.65	1.60	1.05 to 2.45
**Incident involving the child (baseline: no child incident)**	4.29	2.68 to 6.85	3.07	1.48 to 6.35
**Incident involving equipment (baseline: no equipment incident)**	1.37	0.92 to 2.02	1.15	0.75 to 1.74
**Incident involving the vehicle (baseline: no vehicle incident)**	0.76	0.28 to 2.06	0.47	0.23 to 0.96

^a^Adjustments made for: age; PIM2 score; diagnosis of the child; whether the child was ventilated at the time of referral; whether the child was receiving critical care and the time taken to reach the bedside. Cluster term included for the PCCT

## Discussion

In this study, we considered the impact of the care provided during paediatric critical care transport in terms of team composition, the extent of stabilisation and interventions performed, and the occurrence of critical incidents. We found that transports led by ANPs and Junior Doctors resulted in similar outcomes for children and the occurrence of a critical incident during transport, particularly one that affected the child, was associated with poorer patient outcomes. Whilst we did see elevated mortality for children who received prolonged stabilisation compared to those who did not, we believed this was due to residual confounding rather than a true difference and with additional adjustment this difference would likely be attenuated.

Currently, no national guidance exists about the selection of a transport team leader for PCCTs, or whether transports should be triaged to different team leaders depending on the sickness of the child. Therefore, over the years, PCCTs have evolved dynamically in response to the resources available. Decision making regarding team composition is variable across UK PCCTs. In general, many UK PCCTs have relatively fixed team composition (i.e. they do not vary their team composition in individual cases based on the child’s acuity, other than to add a Consultant for sicker cases); however, at a service level, the standard team composition varies depending on whether ANPs and trained Junior Doctors (fellow) are available to staff the team [7]. Outside England and Wales, transport triage scores have been used, mostly to decide whether a physician should accompany the transport team based on the predicted need for interventions, although the evidence base for this approach is weak [12, 13]. Evidence from neonatal transport suggests that ANP-led or Junior Doctor-led transports have similar patient outcomes [14, 15]. We found there were no differences in mortality between ANP-led and Junior Doctor-led paediatric transports. Consultant-led transports had the highest mortality potentially indicating that Consultants were being triaged for sicker patients. The continued slightly elevated mortality may indicate that our adjustment did not fully account for the severity of illness of the patient, as concluded by similar studies [16]. We were unable to assess if Consultants were supporting the training of new doctors or nurses. Although the team leader grade does not appear to be associated with clinical outcomes, future work will need to consider the cost-effectiveness of different team leader models.

Previous research from a single London-based PCCT has suggested that there is no association between the time spent stabilising the child and 24-h mortality [17]. However, in our work we found a difference in mortality between children who received prolonged stabilisation and those who did not. This marked difference diminished substantially after adjustment and we theorise that the remaining adjustment may be due to residual confounding, although future work needs to consider this carefully. More broadly, in previous work, we have shown that the use of specialist transport teams improves patient outcome [5] although other research has demonstrated similar outcomes irrespective of the type of transport team [18]. We have also shown that the time taken for the transport team to reach the bedside of the child in the referring hospital does not appear to impact on mortality [10].

Larger differences did persist for secondary healthcare outcomes, with those children requiring prolonged stabilisation having longer LOS and LOV. These differences may be due to unmeasured confounding, as the PIM2 score [11] was created to account for mortality rather than other outcomes. Alternatively these differences could represent a true difference, indicating that keeping children away from PICU for longer, by taking longer to prepare them for transport, means they require longer to achieve clinical stability within PICU.

The total interventions provided to the child by the referring hospital and the PCCT were a proxy for the sickness of the child, as seen by the marked increase in mortality as the number of interventions increased before our case-mix adjustment. When considering the percentage of those interventions which were delivered by the PCCT, there was no association with mortality as the PCCT delivered a higher percentage of the total interventions. This supports our tentative conclusion that it is safe for PCCTs to take the necessary time to provide the child with the interventions they require before transport. This is likely to be because the referring hospital and the PCCT are working together, from around the time of the request to transport the child to PICU, to provide the child with the most important intensive care interventions that they will ultimately require on the PICU.

We found that critical incidents involving the child (odds ratio: 3.07) or equipment (odds ratio: 1.15) were associated with increased odds of mortality even after adjustment, although the confidence interval for equipment contained one (point of no difference). The incidents involving the vehicle led to reduced odds of mortality, although these events are very uncommon and so this result should not be over-emphasised. We suggest that improving the safety of the transport should be a key quality improvement area for PCCTs. We recommend that all critical incidents, particularly those involving the child, should be reviewed after occurrence.

### Strengths and limitations

Our study represents the first large national study to investigate the impact of care provided around the time of transport to PICU. The data were provided from a linked data source, with PICANet providing the majority of the information. PICANet is a national clinical audit with high-quality data with complete coverage of all PICUs and PCCTs in England and Wales. Submission of clinical data is required within three months of a referral, transport or admission [8].

Whilst we selected our adjustment carefully, residual confounding is likely to still exist. To show the impact of our adjustment on our outcomes we chose to present the unadjusted and adjusted results. Confounding may be a particular issue when considering our secondary outcomes of LOS and LOV as the adjustment for morbidity, specifically the PIM2 score [11], was developed for mortality and has not been validated for use in predicting other outcomes. Prospective studies in this area may be needed to explore this in detail further. We did not investigate the impact of our results on any particular subgroups of children, and it may be that our findings are more important for children of specific characteristics.

We selected our primary outcome to be mortality within 30 days of admission to PICU. This was to allow us to capture information about deaths which occur in other locations, including at home or in hospices. However, upon admission to PICU the child will experience a range of interventions and care and it is clinically unlikely that the effect of the transport will persist long term, hence we investigated other mortality end-points.

### Future work

In this analysis we have only considered children who were transported to PICU and we have not compared with children who were admitted directly to PICU; our immediate future work plans to investigate this. Whilst we have considered the outcomes of children, we have not investigated the cost of providing the PCCT service, for example whether use of a Consultant rather than an ANP is cost effective.

We have not considered the experience and perceptions of parents and children whilst they are preparing for transport to PICU, and during the transport itself. Parents have described the transport to PICU as “the worst journey of our lives” [19]. Other workstreams of the DEPICT Study are considering these experiences separately.

## Conclusion

We have provided evidence that taking time to stabilise critically ill children before transport does not appear to have a negative impact on their outcome. However, critical incidents, especially those specifically involving the child had an impact on mortality. We have also demonstrated that use of a specific team composition do not necessarily confer an advantage in terms of outcome. The dynamic way in which PCCTs have adapted to meet the challenges of their region, both in terms of patient profile and service funding, have not, in terms of team composition, led to a detrimental impact on the outcomes of critically-ill children.

## Supplementary Information

**Additional file 1.**

## Data Availability

The data that support the findings of this study are available from PICANet, ICNARC or NHS Digital but restrictions apply to the availability of these data, which were used for the current study, and so are not publicly available. Data can be requested from PICANet, ICNARC or NHS Digital.

## Abbreviatoins

PCCT: Paediatric Critical Care Transport team
PICU: Paedaitrc Intensive Care Unit
ANP: Advanced Nurse Practitioner
NHS: National Health Service
