# Supplementary material for: The effect of care provided by paediatric critical care transport teams on mortality of children transported to paediatric intensive care units in England and Wales: a retrospective cohort study
Source: BMC Pediatr. 2021 May 3;21:217. doi: 10.1186/s12887-021-02689-x (PMC8089132; doi:10.1186/s12887-021-02689-x)
Supplement: Supplementary file 1 — Additional file 1. [file 12887_2021_2689_MOESM1_ESM.docx]

**The effect of care provided by paediatric critical care transport teams on mortality of children transported to paediatric intensive care units in England and Wales: a retrospective cohort study**

Sarah E Seaton PhD^1^*, Elizabeth S Draper PhD^1^, Christina Pagel PhD^2^, Fatemah Rajah MD^3^, Jo Wray PhD^4^, Padmanabhan Ramnarayan MD^5,6^ on behalf of the DEPICT Study Team^7^

1: Department of Health Sciences, University of Leicester, Leicester, UK, LE1 7RH

2: Clinical Operational Research Unit, University College London, London, UK

3: Yorkshire and Humber Infant and Children’s Transport Service (Embrace), Barnsley, UK

4: Heart and Lung Directorate, Great Ormond Street Hospital for Children NHS Foundation Trust, London, UK

5: Children’s Acute Transport Service (CATS), Great Ormond Street Hospital NHS Foundation Trust, London, UK

6: Respiratory, Critical Care and Anaesthesia Section, Infection, Immunity and Inflammation Research & Teaching Department, UCL GOS Institute of Child Health, London, UK

7: The DEPICT Study team can be contacted via the Principal Investigator on: [pramnarayan@nhs.net](mailto:pramnarayan@nhs.net). The DEPICT Study members are: Victoria Barber, Robert Darnell, Patrick Davies, Elizabeth S Draper, Laura Drikite, Matthew Entwistle, Ruth Evans, Emma Hudson, Enoch Kung, Will Marriage, Stephen Morris, Paul Mouncey, Christina Pagel, Anna Pearce, Eithne Polke, Fatemah Rajah, Padmanabhan Ramnarayan, Sarah E Seaton, Jo Wray.

*Corresponding author: Sarah E Seaton, Department of Health Sciences, University of Leicester, LE1 7RH. Email: [sarah.seaton@leicester.ac.uk](mailto:sarah.seaton@leicester.ac.uk), Tel: 0116 2525434

**APPENDIX**

**Appendix Figure 1:** Flow chart of included/excluded children.


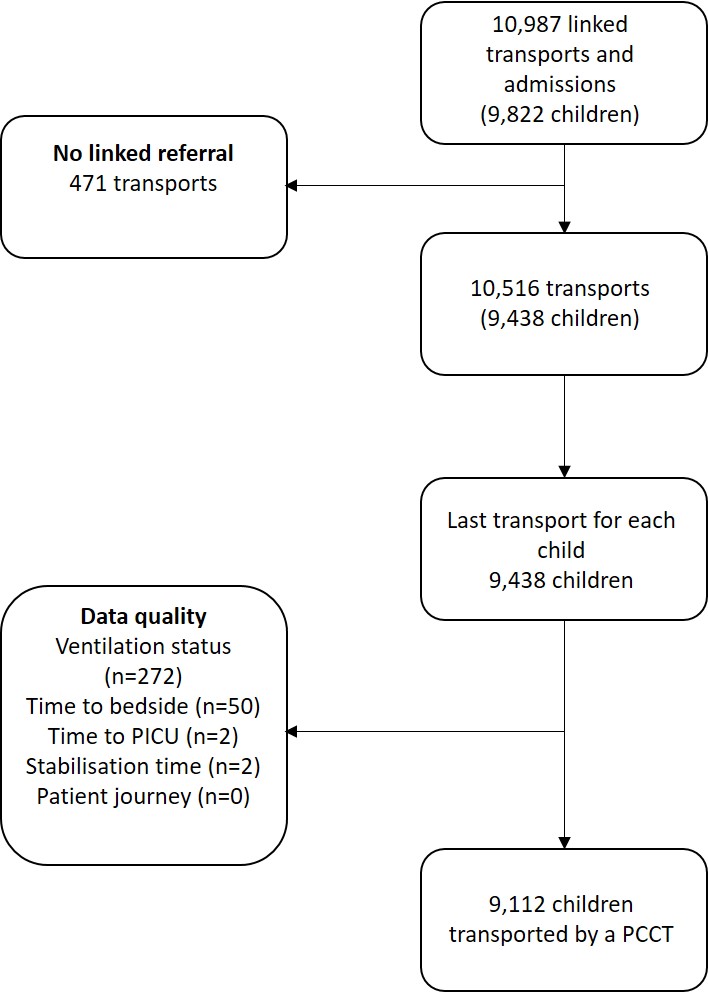


**Appendix Table 1**: Average change in stabilisation time (in minutes) by intervention after adjustment for other factors

| **Characteristic** | **Minutes change in stabilisation time (minutes)** | **95% CI** |
| --- | --- | --- |
| **Intervention conducted whilst PCCT in attendance** |  |  |
| Specified intervention not provided | Baseline | Baseline |
| Intubation | 35.9 | 32.7 to 39.1 |
| Central venous access | 41.4 | 37.8 to 44.9 |
| Arterial access | 26.2 | 23.2 to 29.2 |
| Intraosseous | 41.8 | 34.3 to 49.2 |
| Vasoactive infusion | 22.2 | 19.1 to 25.4 |
| **Age group** |  |  |
| <1 year | Baseline | Baseline |
| 1 to <5 years | -1.2 | -3.8 to 1.3 |
| 5 to <11 years | 3.0 | -0.3 to 6.2 |
| 11 to <16 years | 6.5 | 2.7 to 10.2 |
| **PIM2 group** |  |  |
| <1% | Baseline | Baseline |
| 1 to <5% | 11.5 | 8.1 to 15.0 |
| 5 to <15% | 18.8 | 15.1 to 22.5 |
| 15 to <30% | 29.4 | 24.1 to 34.7 |
| 30+% | 29.1 | 23.1 to 35.1 |
| **DEPICT Diagnoses** |  |  |
| Respiratory | Baseline | Baseline |
| Cardio | -15.5 | -18.8 to -12.2 |
| Endocrine | -3.0 | -9.8 to 3.7 |
| Haem/onc | -10.5 | -18.5 to -2.5 |
| Infection | -5.8 | -9.6 to -1.9 |
| Neuro | -11.4 | -14.5 to -8.4 |
| Trauma & accidents | -9.2 | -14.8 to -3.7 |
| Other | -15.0 | -20.0 to -10.0 |
| **Ventilated at referral** |  |  |
| No (not indicated) | Baseline | Baseline |
| Yes | 7.1 | 4.5 to 9.6 |
| No (advised) | 1.2 | -1.6 to 3.9 |
| **Receiving critical care** |  |  |
| No | Baseline | Baseline |
| Yes | 7.9 | 5.3 to 10.6 |
| **Constant** | 84.5 | 81.0 to 88.0 |

**Appendix Table 2:** Stabilisation interventions initiated by the referring hospital or the PCCT and odds of 30-day mortality after adjustment for other factors

| **Characteristic** | **Odds ratio** | **95% Confidence interval** |
| --- | --- | --- |
| **Intubation** |  |  |
| Not provided | Baseline | Baseline |
| Initiated by local team | 0.65 | 0.43 to 0.97 |
| Initiated by transport team | 0.67 | 0.45 to 1.00 |
| **Vascular access** |  |  |
| Not provided | Baseline | Baseline |
| Initiated by local team | 1.38 | 0.96 to 1.97 |
| Initiated by transport team | 1.20 | 0.78 to 1.85 |
| **Vasoactive infusions** |  |  |
| Not provided | Baseline | Baseline |
| Initiated by local team | 1.67 | 1.37 to 2.05 |
| Initiated by transport team | 1.18 | 0.89 to 1.55 |
| **Time to bedside** |  |  |
| ≤60 minutes | Baseline | Baseline |
| 61-90 minutes | 1.07 | 0.88 to 1.30 |
| 91-120 minutes | 0.85 | 0.65 to 1.13 |
| 121-180 minutes | 1.04 | 0.85 to 1.26 |
| 181+ minutes | 0.79 | 0.63 to 1.00 |
| **Age group** |  |  |
| <1 year | Baseline | Baseline |
| 1 to <5 years | 0.91 | 0.76 to 1.09 |
| 5 to <11 years | 1.29 | 1.05 to 1.58 |
| 11 to <16 years | 1.12 | 0.86 to 1.47 |
| **PIM2 group** |  |  |
| <1% | Baseline | Baseline |
| 1 to <5% | 2.21 | 1.13 to 4.32 |
| 5 to <15% | 3.44 | 1.74 to 6.80 |
| 15 to <30% | 10.05 | 4.83 to 20.91 |
| 30+% | 28.35 | 13.92 to 57.75 |
| **Diagnoses** |  |  |
| Respiratory | Baseline | Baseline |
| Cardiovascular | 2.02 | 1.30 to 3.14 |
| Endocrine | 2.42 | 1.62 to 3.62 |
| Haem/onc | 2.32 | 1.08 to 4.99 |
| Infection | 1.40 | 0.92 to 2.13 |
| Neurological | 1.34 | 0.81 to 2.21 |
| Trauma and accidents | 1.24 | 0.87 to 1.77 |
| Other | 1.65 | 0.85 to 3.19 |
| **Ventilated at referral** |  |  |
| No | Baseline | Baseline |
| Yes | 1.40 | 1.15 to 1.72 |
| No (advised to ventilate) | 0.98 | 0.75 to 1.27 |
| **Receiving critical care** |  |  |
| No | Baseline | Baseline |
| Yes | 0.96 | 0.82 to 1.12 |

Note: cluster term for the PCCT is included

**Appendix Figure 2a**: Unadjusted and adjusted expected length of stay by the percentage of interventions delivered whilst the PCCT were present and in total. Adjusted probabilities are estimated whilst holding other covariates at their average value. Note children receiving no interventions at all are in the <20% category.

Adjustments were made for: time taken to reach the bedside of the child; age of child; PIM 2 score; diagnosis of the child; whether they were ventilated at the time of referral and whether they were receiving critical care.

**Appendix Figure 2b:** Unadjusted and adjusted expected length of ventilation by the percentage of interventions delivered whilst the PCCT were present and in total. Adjusted probabilities are estimated whilst holding other covariates at their average value. Note children receiving no interventions at all are in the <20% category.

Adjustments were made for: time taken to reach the bedside of the child; age of child; PIM 2 score; diagnosis of the child; whether they were ventilated at the time of referral and whether they were receiving critical care.
